# Does Forgiveness Underlie the Relationship Between Religiosity and Meaning in Life Among Members of Sexaholics Anonymous in Poland?

**DOI:** 10.1007/s10943-023-01842-3

**Published:** 2023-06-09

**Authors:** Marcin Wnuk, Edyta Charzyńska

**Affiliations:** 1 Department of Work and Organizational Psychology, Adam Mickiewicz University in Poznań, Szamarzewskiego 89/AB, 60-568 Poznań, Poland; 2 Faculty of Social Sciences, Institute of Psychology, Institute of Pedagogy, University of Silesia in Katowice, Bankowa 12, 40-007 Katowice, Poland

**Keywords:** Religiosity, Meaning in life, Forgiveness, Sexaholics Anonymous, Poland

## Abstract

Religiosity and meaning in life are recognized as factors supporting recovery from addictions. However, little is known about the moral mechanisms involved in the relationship between religiosity and meaning in life among individuals with addictions. The main purpose of this study was to test the direct and indirect (through forgiveness by God/higher power and interpersonal forgiveness) relationships between subjective religiosity and the presence of meaning in life among 80 members (72 men and 8 women) of Sexaholics Anonymous (SA) in Poland. The following measures were used: a single-item measure of subjective religiosity, subscales from the Forgiveness Scale and the Heartland Forgiveness Scale, and the Meaning in Life Questionnaire. The sequential mediation model was tested using Hayes PROCESS macro. The results showed a direct positive relationship between subjective religiosity and the presence of meaning in life. Moreover, subjective religiosity was positively related to forgiveness by God/higher power, which, in turn, directly and indirectly (through interpersonal forgiveness) predicted higher levels of the presence of meaning in life. The study suggests that among SA members, religious faith facilitates perceiving one’s own life as meaningful, both directly and indirectly, through aspects of forgiveness. Members of SA may benefit from their belief in God/higher power and religiously-rooted forgiveness to support the meaning-making processes.

## Introduction

### Compulsive Sexual Behavior Disorder and Self-Help Groups for Its Treatment

In the research literature, having repetitive and intrusive sexually-related thoughts, urges, and behaviors that maintain over an extended period (e.g., six months or more) and cause significant psychosocial distress and functional impairment are termed compulsive sexual behavior disorder (CSBD) (also known as hypersexuality, sexual addiction, or sexual impulsivity; Kowalewska & Lew-Starowicz, 2021). This problem affects about 3–6% of the adult population (Sassover & Weinstein, 2020). CSBD is officially included as a new entity in the 11th edition of the *International Classification of Diseases* (ICD-11) (World Health Organization, 2018). Although the ICD-11 classifies CSBD as an impulse control disorder, many similarities have been noted between CSBD and substance and behavioral addictions in terms of neurobiological processes, co-occurring psychiatric disorders, psychosocial predictors, and therapeutic approaches (Kafka, 2010; Potenza et al., 2017).

One of the options for treating CSBD is joining a self-help group. Historically, the Sex and Love Addicts Anonymous (SLAA; Augustine Fellowship & SLAA, 1986) was the first group of this type dedicated to individuals engaging in uncontrolled fantasies, urges, and behaviors. Nowadays, in the US, apart from the SLAA, several other self-help groups for treatment of CSBD are available: Sexaholics Anonymous (SA), Sex Addicts Anonymous (SAA), Sexual Compulsives Anonymous (SCA), and Sexual Recovery Anonymous (SRA). In Poland, individuals with CSBD can seek support in SA and SLAA.

All self-help groups for individuals with CSBD are based on a twelve-step program adapted from Alcoholics Anonymous (AA, 1972), which treats addiction as an incurable and progressive disease of the body, mind, and spirit (Kurtz, 1982). Self-help groups for CSBD posit that admitting one’s own inability to control own sexual desires and behaviors and surrendering to God/higher power are the requirements for the recovery process (SA, 1989). During the self-help group meetings, individuals learn how to deal with their own sexual phantasies and urges, overcome existential emptiness, gain more self-awareness, and rebuild relationships with others based on moral virtues (Augustine Fellowship & SLAA, 1986; SA, 1989). Self-help groups for CSBD share similar assumptions and objectives (Efrati & Gola, 2018), whereas the main difference between them lies in their ways of defining sexual abstinence, with SA being the most restrictive in this regard, allowing only conjugal, heterosexual sex with one’s spouse (SA, 1989).

### Meaning in Life and Addiction

The meaning in life as a eudaimonic facet of well-being refers to the degree to which an individual perceives their life as significant, purposeful, and valuable (Steger et al., 2006). According to some existential theories, an addiction may stem from experiencing existential emptiness, apathy, and unsuccessful attempts to fulfill the human’s natural need for spiritual growth (Coleman et al., 1986; Frankl, 1992). Moreover, the progression of addiction may exacerbate these experiences and feelings, resulting in continual failure to find meaning in life and a chronic and pervasive sense of hopelessness and despair.

Consistent with these theoretical premises, studies have demonstrated a negative relationship between the sense of meaning in life and substance use and misuse and behavioral addictions (Hu et al., 2022; Laudet et al., 2006; Zhang et al., 2015). Moreover, research has supported the positive role of meaning in life for objective (abstinence and recovery) and subjective (well-being) outcomes of treatment of addictions (DeLucia et al., 2016; Gutierrez, 2019; Laudet et al., 2006; Lyons et al., 2011). Despite these results, there are still very few studies on the predictors of meaning in life and the mechanisms involved in this relationship in samples of individuals with addictions. In the current study, we attempt to fulfill this gap by examining the direct and indirect (through forgiveness) relationships between subjective religiosity and the presence of meaning in life among SA members.

### Religion as a Meaning System

Religion performs several functions crucial for human well-being, both in everyday life and in times of adversity. It is a very important source of support (Koenig et al., 2001) and a coping resource (Klaassen et al., 2006). It also promotes positive emotions (Koenig et al., 2001; Park, 2007) and healthy behaviors (Cohen & Koenig, 2003). In addition, religion provides individuals with a means through which they can experience a sense of meaning in their lives (Wnuk & Marcinkowski, 2014). Religion as a meaning system constitutes a structure by which religious individuals perceive, evaluate, understand, and interpret the world and the events taking place around them as an element of something larger and more significant than their own existence, in this way, organizing and orienting their behaviors (Silberman, 2005).

A large number of studies demonstrated that religiosity is positively related to finding meaning and purpose in life (Prinzing et al., 2021; Steger et al., 2006; Vilchinsky & Kravetz, 2005). Despite the presumably high importance of the topic of meaning in life among individuals with addictive behaviors, there is a lack of data on the relationship between subjective religiosity and the presence of meaning in life in the sample of individuals attending the SA meetings. Based on the findings of previous research in the addiction field (Gutierrez, 2019; Laudet et al., 2006; Lyons et al., 2011; Wnuk, 2015), supporting a positive relationship between religiosity and meaning in life, we hypothesize that:

#### Hypothesis 1 (H1)

In the sample of SA members, there is a direct positive relationship between subjective religiosity and the presence of meaning in life.

### Religiosity and Meaning in Life: Forgiveness as a Potential Mediator

Besides having a direct positive relationship with the presence of meaning in life, religiosity may also be indirectly related to it through forgiveness. Many religions treat forgiveness as one of the most important and desirable attributes that build the axiological systems of religious people (Shults & Sandage, 2003). Consistent with this, studies have shown that religiosity is positively related to forgiveness (Davis et al., 2013; Kane et al., 2021; Stewart et al., 2016).

Besides the correlational relationships, there is also some evidence regarding the causal effect of religiosity on forgiveness (Fincham & May, 2021). For example, Vasiliauskas and McMinn (2013) conducted an experimental intervention among undergraduate students from private Christian colleges to examine the role of prayer in forgiving an interpersonal offense. Compared to the control group, the prayer group showed a significant increase in the state of forgiveness.

A disposition to forgive helps a person stop negative responses to a transgressor (Davis et al., 2013). Learning this moral virtue seems to be important in individuals with CSBD since this group demonstrates higher scores on early life trauma. More specifically, symptoms of CSBD have been found to correlate positively with emotional neglect, emotional abuse, parentification, sexual harassment, physical abuse, and sexual abuse (Efrati & Gola, 2019). These findings suggest that many individuals with CSBD may have been emotionally, psychologically, and sexually hurt by their parents and other significant others, which may evoke negative emotions such as hate, anger, or sadness, with which they have to cope as adults (Kowalewska & Lew-Starowicz, 2021).

In the situation of interpersonal transgression, the forgiveness disposition motivates a harmed person to reinterpret and redefine what has happened, which may mitigate negative emotions toward the transgressor and facilitate the process of finding meaning in the experience of harm (Park, 2007). Consistent with these theoretical premises, many correlational studies showed that forgiveness is positively related to finding meaning and purpose in life (Allan, 2015; Głaz, 2019; Krok & Zarzycka, 2021). Moreover, in a six-month, longitudinal study of romantic couples, Van Tongeren et al. (2015) noted that participants who regularly forgave their partner declared increased meaning in life over time, which suggests a causal relationship between forgiveness and meaning in life.

### Forgiveness by God/Higher Power and Interpersonal Forgiveness

The results of the aforementioned studies suggest that a disposition to forgive may underlie the relationship between religious faith and meaning in life. This assumption is also supported by a study by Lyons et al. (2011) carried out on a sample of Australian individuals dependent on various substances and participating in a therapeutic program. In this study, the forgiveness of self and receiving forgiveness from others and God mediated the relationship between spiritual experiences and purpose in life.

In the current study, we focus on this aspect of forgiveness, which is very closely related to religion—namely, forgiveness by God (see Huber, Allemand & Huber, 2011; Krause, 2015), which potentially precede other aspects of forgiveness among religious individuals. Forgiveness by God involves recognizing that God has forgiven faults and other behaviors that the individual perceives as morally wrong (Toussaint et al., 2001). This construct may be especially important for members of self-help groups for addictions who accept their powerlessness over the addiction and believe that the help of a power greater than themselves is needed to restore them to sanity (AA, 1972; SA, 1989). In this study, we used the term “forgiveness by God/higher power” to make the construct better adjusted to the premises of SA, according to which a belief in a higher power does not have to be connected with any religious denomination (Augustine Fellowship & SLAA, 1986; SA, 1989).

Based on the above rationales, in the current study, we expect that:

#### Hypothesis 2 (H2)

In the sample of SA members, subjective religiosity is positively related to forgiveness by God/higher power, which, in turn, predicts higher levels of the presence of meaning in life.

The second aspect of forgiveness investigated in this study was interpersonal forgiveness (also called “forgiveness of others”). Based on previous studies that noted positive relationships between religiosity and interpersonal forgiveness (Davis et al., 2013; Kane et al., 2021; Rye & McCabe, 2014) and between interpersonal forgiveness and meaning in life (Krok & Zarzycka, 2021; Van Tongeren et al., 2015), we expected that:

#### Hypothesis 3 (H3)

In the sample of SA members, subjective religiosity is positively related to interpersonal forgiveness, which, in turn, predicts higher levels of the presence of meaning in life.

Importantly, the feeling that one receives God’s/higher power’s mercy may promote a deliberate, intentional process involving the reduction of negative responses to an offender (Davis et al., 2013). Many studies demonstrated the positive relationship between forgiveness by God and interpersonal forgiveness (Chen et al., 2019; Hirsch et al., 2012; Huber et al., 2011). There is also some evidence that the feeling of being forgiven by God predicts interpersonal forgiveness but not vice versa. For instance, in two recent studies, Fincham and May (2021) investigated a potentially bidirectional relationship between divine forgiveness and interpersonal forgiveness. Study 1 showed that divine forgiveness predicted interpersonal forgiveness seven weeks later. By contrast, the predictive role of interpersonal forgiveness for divine forgiveness was not observed. The results were replicated in study 2, in which the authors used a longer interval (14 weeks) and accounted for socially desirable responding (Fincham & May, 2021).

Taking all this together, we formulate the following hypothesis, including the sequential relationships between religiosity, forgiveness by God/higher power, interpersonal forgiveness, and the presence of meaning in life:

#### Hypothesis 4 (H4)

In the sample of SA members, subjective religiosity is positively related to feeling forgiven by God/higher power, which, in turn, indirectly, through interpersonal forgiveness, predicts higher levels of the presence of meaning in life.

## Methods

### Participants

The demographic characteristics of the participants are presented in Table 1. The study was conducted among 80 individuals (90% of men and 10% of women) attending SA meetings in Poland. The average age of the participants was 38.96 years (SD = 10.56). Most participants graduated from universities (78.8%), were unmarried (51.3%), and declared to be Roman Catholic (82.5%).

**Table 1:** Participant demographic characteristics

Characteristic	Categories	n (%)
Gender	Men	72 (90%)
	Women	8 (10%)
Age (years)	22–29	13 (16.2%)
	30–37	28 (35.0%)
	38–45	18 (22.5%)
	46–53	11 (13.8%)
	54–61	9 (11.3%)
	62–68	1 (1.2%)
Education	Vocational	2 (2.4%)
	Secondary	15 (18.8%)
	Higher	63 (78.8%)
Marital status	Single	41 (51.3%)
	Married	36 (45%)
	Separated	2 (2.5%)
	Divorced	1 (1.2%)
Religion	Roman Catholicism	66 (82.5%)
	Jehovah’s Witnesses	2 (2.5%)
	Slavic religion	2 (2.5%)
	Without denomination	9 (11.2%)
	Agnostic	1 (1.3%)
Previous use of psychological or therapeutic services	No	6 (7%)
	Yes	74 (93%)
Current use of psychological or therapeutic services	No	44 (55%)
	Yes	36 (45%)
Number of months in SA (M ± SD)	47.48 (32.86)	
Number of months of sexual abstinence (M ± SD)	20.95 (28.64)	
Number of the steps completed (M ± SD)	6.99 (4.17)	

*M* mean, *SD* standard deviation. *N* = 80

On average, the participants had been attending SA meetings for nearly 4 years (*M* = 47.48 months; SD = 32.86), maintaining sexual abstinence from acting out for more than 1.5 years (*M* = 20.95 months; SD = 28.64), and completed 7 of the 12 steps (*M* = 6.99; SD = 4.17). Nearly half of the participants (45.0%) were currently using psychological or therapeutic services.

### Measures

#### Subjective Religiosity

Subjective religiosity was measured with a single item: “To what extent do you consider yourself to be religious?”. Participants responded using a 5-point Likert scale ranging from 1 = “not at all religious” to 5 = “very religious.” Single-item measures of subjective religiosity have been used in many studies, which supported their reliability and validity (Abdel-Khalek, 2007; Gorsuch & McPherson, 1989).

#### Forgiveness by God/Higher Power

To measure the level of feeling forgiven by God/higher power, we used a subscale from the Forgiveness Scale, which is a Polish adaptation (Charzyńska & Heszen, 2013) of forgiveness indices by Toussaint et al. (2001). The participants were asked to respond to two statements: “Knowing that I am forgiven for my sins gives me the strength to face my faults and be a better person.” and “I know that God/higher power forgives me.” The responses were given on a 5-Likert scale ranging from 1 (“strongly disagree”) to 5 (“strongly agree”). The additional “not applicable” option was included because some respondents could not have believed in God/higher power. The total score was calculated by summing up the responses to the two items. In this study, the reliability of the measure assessed using Cronbach’s *α* was 0.82.

#### Interpersonal Forgiveness

The disposition to interpersonal forgiveness was measured using the Forgiveness of Others subscale from the Polish adaptation (Kaleta et al., 2016) of the Heartland Forgiveness Scale (Thompson et al., 2005). The subscale consists of six items (example item: “Although others have hurt me in the past, I have eventually been able to see them as good people.”) that are assessed with a 7-point Likert scale ranging from 1 = “Almost always false of me” to 7 = “Almost always true of me.” Three items were recoded before calculating the total score. In the current study, the internal consistency of the Forgiveness of Others subscale was *α* = 0.66.

#### Presence of Meaning in Life

The presence of meaning in life was assessed with the subscale taken from the Polish adaptation (Kossakowska et al., 2013) of the Meaning in Life Questionnaire (Steger et al., 2006). The participants responded to each of the five items (e.g., “I have discovered a satisfying life purpose.”) using a 7-point Likert scale, ranging from 1 = “absolutely untrue” to 7 = “absolutely true.” The total score was derived by summing up the responses to all items. In the current study, Cronbach’s α for the Presence of Meaning in Life subscale of the MLQ was 0.90.

### Procedure

The study was performed in line with the principles of the Declaration of Helsinki. Approval was granted by the Ethics Committee at the University of Silesia in Katowice (KEUS 123/05.2021). The snowball sampling was used to reach potential participants. The research team member [MW] contacted by e-mail several SA members with whom he was familiar and asked them to disseminate the information about the study among other SA members. The e-mail describing the study included its purpose, the link to the survey, and the names of the researchers.

After going to the survey website, each participant read the introductory note, which informed them about the research purposes, anonymous and voluntary participation in the study, the approximate duration of the survey, and the right to withdraw from the research at any time. After reading the instruction, each participant was asked to give their consent to participate in the study by checking the appropriate box. No incentives or compensation for participation were offered.

### Statistical Analyses

In the first step of the analysis, we performed Harman’s single factor test to exclude potential common method bias (Podsakoff et al., 2003). If the total variance extracted by one factor exceeds 50%, then common method bias may be present. In the next step, we calculated the means, standard deviations, and zero-order correlations between the variables. The calculations were conducted using IBM SPSS version 27.0 (IBM Corp., 2020).

To test the hypotheses, we used Model 6 of the PROCESS macro developed by Hayes (2013). The significance of the indirect effects was tested using the bootstrapping method with 5000 subsamples and 95% bootstrap confidence intervals; the parameter is considered significant if the confidence interval does not include zero. The effect size for the specific indirect effect was calculated as a completely standardized indirect effect (Kenny, 2021; Preacher & Kelley, 2011). Moreover, we added “the current use of psychological or therapeutic services” (dummy-coded as 0 = “no” and 1 = “yes”) to the model, treating it as a control variable (by using the “Covariate(s)” box in the PROCESS macro). We did so taking into account that the topics of forgiveness and meaning in life can be discussed during therapeutic sessions (Akhtar & Barlow, 2018; Hill et al., 2017), and therefore the current use of psychological or therapeutic services may reduce unexplained variation in forgiveness by God/higher power, interpersonal forgiveness, and the presence of meaning in life.

## Results

### Preliminary Analysis

Harman’s single factor test showed that the single factor explained 37.1% of the variance, which was lower than the threshold of 50%. This suggests that the results of our study were not affected by the common method bias (Podsakoff et al., 2003).

Table 2 presents the descriptive statistics and bivariate correlations between the study variables. Subjective religiosity, forgiveness by God/higher power, and interpersonal forgiveness were positively related to the presence of meaning in life. Moreover, subjective religiosity was positively related to forgiveness by God/higher power, but it was unrelated to interpersonal forgiveness. In addition, forgiveness by God/higher power was positively related to interpersonal forgiveness and negatively related to the current use of psychological or therapeutic services.

**Table 2:** Descriptive statistics and bivariate correlations between the study variables

Variables	(1)	(2)	(3)	(4)	(5)
(1) Subjective religiosity	1				
(2) Forgiveness by God/higher power	.32**	1			
(3) Interpersonal forgiveness	.09	.46***	1		
(4) Presence of meaning in life	.36**	.54***	.50***	1	
(5) Current use of psychological or therapeutic services	− .01	− .27*	− .09	− .04	1
M	3.76	8.69	29.06	25.63	45%^a^
SD	1.22	1.58	5.06	6.47	–
Range	1–5	3–10	16–39	10–35	–

^a^The percentage of the participants currently using psychological or therapeutic services

The current use of psychological or therapeutic services was dummy-coded (0 = “no,” 1 = “yes”). *p < .05, **p < .01, ***p < .001. *M* = mean, *SD* = standard deviation

### Sequential Mediation Model

**Fig. 1:**
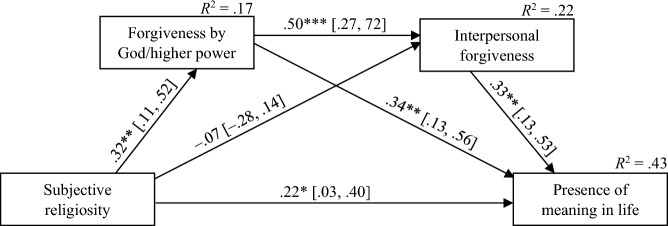
Sequential Mediation Analysis Results. *Note*. The path coefficients are presented in their standardized form (β); numbers in brackets represent the 95% bootstrap confidence intervals. *p < .05, **p < .01, ***p < .001. *R*^2^ = explained variance. For clarity, the control variable (current use of psychological or therapeutic services) was omitted in Fig. 1. The control variable was negatively related to forgiveness by God/higher power (β =  − .27, 95% CI [− .47, − .06]; *p* = .013)

Figure 1 presents the standardized parameters for the sequential mediation model. The total effect of subjective religiosity on the presence of meaning in life was significant (β = 0.36; 95% CI [0.14, 0.57]; *p* = 0.001). It comprised a significant direct effect (β = 0.22; 95% CI [0.03, 0.40]; *p* = 0.022) and the significant total indirect effect (β = 0.14; 95% CI [0.01, 0.30]).

As for specific indirect effects, the significant indirect effect of subjective religiosity on the presence of meaning in life through forgiveness by God/higher power was found, with the value of the coefficient and its confidence intervals suggesting a small to medium effect size (β = 0.11, 95% CI [0.01, 0.25]). The specific indirect effect of subjective religiosity on the presence of meaning in life through interpersonal forgiveness was insignificant (β =  − 0.02, 95% CI [− 0.09, 0.03]).

The most complex indirect effect tested involved the cascading effect of subjective religiosity on the presence of meaning in life through the sequential path of forgiveness by God/higher power—interpersonal forgiveness. This effect turned out to be significant (β = 0.05, 95% CI [0.01, 0.12]) and can be considered low to medium. Specifically, subjective religiosity was positively related to forgiveness by God/higher power (β = 0.32, 95% CI [0.11, 0.52]; *p* = 0.003), which, in turn, was positively related to interpersonal forgiveness (β = 0.50, 95% CI [0.27, 0.72]; *p* < 0.001), predicting higher levels of the presence of meaning in life (β = 0.33, 95% CI [0.13, 0.53]; *p* = 0.001). The proportion of variance explained (*R*^2^) was 0.17 for forgiveness by God/higher power, 0.22 for interpersonal forgiveness, and 0.43 for the presence of meaning in life.

## Discussion

### Direct and Indirect Relationships Between Religiosity and Meaning in Life

The purpose of the study was to examine the direct and indirect relationships between subjective religiosity and the presence of meaning in life among individuals attending SA meetings in Poland. As a potential mediator of these relationships, we considered forgiveness, which is a moral virtue theoretically and empirically related to religiosity and meaning in life. Instead of taking the approach in which general levels of this moral virtue would be in the spotlight, we focused on forgiveness by God/higher power, which we treated as a potential source of interpersonal forgiveness. We took this perspective following the results of studies conducted on various samples, including individuals with addictions, which showed that different aspects of forgiveness might be shaped by different predictors and lead to different consequences (Charzyńska, 2021; Hirsch et al., 2012).

Consistent with H1, the direct relationship between subjective religiosity and the presence of meaning in life was significant. This result corroborates the findings of previous studies, which showed that religiosity facilitates meaning in life (Prinzing et al., 2021; Steger et al., 2006). It suggests that for religious SA members, religion facilitates finding a meaning system, which equips them with a set of beliefs and guidelines regarding how to live, from which they can derive a comprehensive framework for building personal meaning based on the ultimate meaning (Silberman, 2005; Wnuk & Marcinkowski, 2014).

As expected, the SA members’ level of subjective religiosity was related to the feeling of being forgiven by God / a higher power, which, in turn, predicted the presence of meaning in life (H2 supported). Similar results were noted in a study by Fincham and May (2020), in which religious involvement was positively related to divine forgiveness, which, in turn, predicted depressive symptoms among college students. Moreover, in a sample of drug-addicted individuals participating in self-help groups, divine forgiveness mediated the relationship between daily spiritual experiences and meaning in life (Lyons et al., 2011). Contrary to our expectations, after controlling for other variables in the model, the indirect relationship between subjective religiosity and the presence of meaning in life through interpersonal forgiveness was not supported in the present study (H3 rejected).

Based on recent empirical findings showing that God’s forgiveness predicts interpersonal forgiveness over time but not vice versa (Fincham & May, 2021), we expected that forgiveness by God/higher power might predict interpersonal forgiveness among SA members. H4, concerning the sequential relationships between subjective religiosity, forgiveness by God/higher power, interpersonal forgiveness, and the presence of meaning in life, was supported in our study. 

The forgiveness mechanism underlying the relationship between religiosity and meaning in life among SA members may be explained by the process of modeling moral virtues by faith (Bandura, 1977). Perceiving God/higher power as forgiving and merciful may encourage SA members to adopt this approach toward other people to follow the path designated for them by the Transcendence. Religious SA members may see forgiving others as a moral imperative, with God/higher power forgiving human wrongdoing establishing an ideal example of what each believer is called for (Fincham & May, 2021; Webb et al., 2005). This is consistent with the results of the study by Tsang et al. (2005), which showed that forgiving and merciful God images were positively associated with forgiving others and negatively with avoidant motivation (see also Webb et al., 2005). The process of changing the perception of God by the members of self-help groups is also included in a twelve-step program, especially Step 3, which supports changing the concept of God from punitive to merciful (Augustine Fellowship & SLAA, 1986; SA, 1989).

In addition, for Roman Catholics, who constituted a large part of our sample, Jesus could serve as a perfect role model of a forgiving attitude (Davis et al., 2013). Feeling God’s forgiveness may also motivate the person who has been harmed to think about themselves not as a victim holding a grudge and refusing to forgive the transgressor but as a person undergoing a test of faith, which may result in making attempts to find meaning in the experience of transgression. This attitude may be accompanied by diminishing the “magnitude” of the harms one had suffered, in contrast to the harm Jesus suffered on the cross.

To summarize, the results of our study can be interpreted considering forgiveness by God/higher power and interpersonal forgiveness as meaning-making mechanisms, which are partially based on one’s religious sources. This religious meaning-oriented system creates the moral-cognitive-spiritual framework to perceive, interpret and integrate situations and events and build attitude toward life and the world as meaningful, purposeful, predictable, comprehensive, and manageable (Głaz, 2019; Krok & Zarzycka, 2021; Silberman, 2005). The additional function of this system is to provide answers to difficult existential questions about the appropriate ways of life, choosing values, suffering, or death.

As Frankl (1992) stated, attitudinal values are the essence of human beings as they give an individual the chance to confront suffering with dignity and the opportunity to find meaning in a painful and difficult life situation. This “tragic optimism,” resulting from a religious meaning-oriented system built upon forgiveness, may allow the SA members to transform their pain related to the lack of control over sexual behaviors into something meaningful and valuable.

### Theoretical and Practical Implications

The present study has three important theoretical implications. First, it supports the significant relationship between SA members’ subjective religiosity and their meaning in life through the path of forgiveness by God/higher power—interpersonal forgiveness. In this way, it corroborates the meaning-making role played by religiosity and forgiveness as a related moral virtue, which in the long term may form recovery capital that advances the capacity of individuals to sustain recovery (Charzyńska, 2021; Lyons et al., 2011).

Second, although our study was cross-sectional, it corresponds with the results of the studies showing that forgiveness by God/higher power may predict interpersonal forgiveness (Fincham & May, 2021; Huber et al., 2011). These results suggest that for religious SA members, moral virtues related to the relationship with God/higher power may be the primary factors, which may then be translated into the horizontal domain of interpersonal relationships, promoting perceiving one’s own life as meaningful and purposeful as a result of restoring relationships with significant others. Third, the findings indicate the need to consider various aspects of forgiveness in studies conducted among members of self-help groups to identify the relationships between these constructs and, in this way, gain a deeper insight into their role in finding meaning in life.

From the practical point of view, counselors, practitioners, and therapists should consider developing workshops, educational programs, and interventions, which may help individuals with CSBD use forgiveness to facilitate the process of finding meaning in their lives. Religiously inclined patients suffering from CSBD could be taught to use their religiosity as a potential source of experiencing forgiveness from God/higher power, which, in turn, could improve their sense of meaning in life by enhancing forgiveness in interpersonal relationships. Such patients could also be encouraged to attend SA meetings as a “spiritual supplement” to professional therapy. For those individuals with CSBD who are religious skeptics, forgiveness interventions could be incorporated into CSBD treatment to develop the disposition toward forgiveness, which may support them in finding meaning in their lives.

### Limitations

Our study has some limitations, which should be discussed. First of all, due to using snowball sampling, we cannot calculate a response rate, which limits the generalizability of the results. Moreover, our sample included mostly well-educated men who declared to be Roman Catholic. Future research involving other self-help groups for individuals with CSBD, and more religiously and demographically diversified samples are warranted.

In addition, researchers may consider investigating potential gender differences in the relationships studied, which was impossible in the current study due to a low number of women in the sample (*n* = 8). It is also recommended to replicate our research in other cultural contexts, especially in more secular countries than Poland. Because the beneficial effect of religion is present mostly in religious nations and countries (Diener et al., 2011; Lun & Bond, 2013), and the attitudes toward sexual behaviors may be more conservative and restrictive in highly religious countries (Heinemann et al., 2016), the results of our study may be country-specific to some extent. However, our model was based on the results of previous studies conducted in different countries, which showed the significant relationships between the variables included in our study.

The study was cross-sectional, which prevents us from drawing unambiguous conclusions about the direction of the relationships between the variables. Although other directions or bidirectional relationships between the variables are possible, it should be highlighted that all paths in our model were well justified by the findings of previous studies. Nevertheless, there is clearly a need to investigate the temporal relationship between the studied variables among members of self-help groups for addictions to provide evidence for cause-and-effect relationships.

In the current study, subjective religiosity was measured by asking participants how much they perceived themselves to be religious. We chose a single-item measure of subjective religiosity to mitigate the participants’ response burden, also considering that the reliability and validity of such short measures of religiosity have been supported in previous studies (Abdel-Khalek, 2007; Gorsuch & McPherson, 1989). Nevertheless, future studies may benefit from using longer measures to assess SA members’ religiosity. Moreover, although subjective religiosity has been frequently used as an indicator of religiosity in previous studies, future research may consider including more specific indicators, such as religious orientations, religious coping, and objective indicators of religiosity, such as the frequency of attendance in religious services or frequency of prayer.

## Conclusion

Our study showed the direct and indirect relationships between religiosity and the presence of meaning in life among SA members. A path involved in the indirect mechanism included forgiveness by God/higher power and interpersonal forgiveness. The findings indicate that among SA members, religious faith may support the meaning-making processes directly and indirectly through religiously-rooted forgiveness. In light of the results, incorporating patients’ religiosity into CSBD treatment can be suggested as a way to develop a psychological resource in the form of forgiveness, which may promote perceiving one’s life as meaningful, presumably supporting recovery from CSBD.
